# Optogenetic stimulation of cholinergic fibers for the modulation of insulin and glycemia

**DOI:** 10.1038/s41598-021-83361-3

**Published:** 2021-02-11

**Authors:** Arjun K. Fontaine, David G. Ramirez, Samuel F. Littich, Robert A. Piscopio, Vira Kravets, Wolfgang E. Schleicher, Naoko Mizoguchi, John H. Caldwell, Richard F. ff. Weir, Richard K. P. Benninger

**Affiliations:** 1 Department of Bioengineering, University of Colorado, Anschutz Medical Campus, Boulder, USA; 2 Department of Cell and Developmental Biology, University of Colorado, Anschutz Medical Campus, Boulder, USA; 3 Biomechatronics Development Laboratory, University of Colorado, Anschutz Medical Campus, Boulder, USA; 4 Barbara Davis Center for Childhood Diabetes – Anschutz Medical Campus, Boulder, USA; 5 Division of Pharmacology, Department of Diagnostic and Therapeutic Sciences, Meikai University School of Dentistry, Saitama, Japan

**Keywords:** Biological techniques, Biotechnology, Neuroscience, Physiology, Diseases

## Abstract

Previous studies have demonstrated stimulation of endocrine pancreas function by vagal nerve electrical stimulation. While this increases insulin secretion, expected concomitant reductions in circulating glucose do not occur. A complicating factor is the non-specific nature of electrical nerve stimulation. Optogenetic tools, however, provide the potential for cell-type specific neural stimulation using genetic targeting and/or spatially shaped excitation light. Here, we demonstrate light-activated stimulation of the endocrine pancreas by targeting parasympathetic (cholinergic) axons. In a mouse model expressing ChannelRhodopsin2 (ChR2) in cholinergic cells, serum insulin and glucose were measured in response to (1) ultrasound image-guided optical stimulation of axon terminals in the pancreas or (2) optical stimulation of axons of the cervical vagus nerve. Measurements were made in basal-glucose and glucose-stimulated conditions. Significant increases in plasma insulin occurred relative to controls under both pancreas and cervical vagal stimulation, while a rapid reduction in glycemic levels were observed under pancreatic stimulation. Additionally, ultrasound-based measurements of blood flow in the pancreas were increased under pancreatic stimulation. Together, these results demonstrate the utility of in-vivo optogenetics for studying the neural regulation of endocrine pancreas function and suggest its therapeutic potential for the control of insulin secretion and glucose homeostasis.

## Introduction

Neural modulation of the endocrine pancreas impacts insulin secretion and glucose homeostasis. Vagus nerve electrical stimulation activates insulin secretion in multiple mammalian animal models^1–6^, an effect which is mediated by parasympathetic (cholinergic) inputs as it is reduced or abolished with acetylcholine receptor antagonists^1,2,4,5,7^. However, electrical stimulation affects not only the pancreas, but also a multitude of internal thoracic and abdominal organs. Optogenetic stimulation of subsets of vagal axons was investigated here as an alternative approach for modulating pancreas function.

Therapies for Type 2 Diabetes include improving insulin sensitivity via biguanides (metformin) or by increasing glucose excretion via SGLT2 inhibitors. Enhancing insulin secretion is also a key goal in treating patients with Type 2 Diabetes^8^. Side effects associated with K_ATP_ inhibitors (hypoglycemia and β-cell apoptosis^8,9^) have limited their current usage. Thus, GLP1R agonists are currently the only therapy for diabetes with a good safety profile that stimulates insulin release^10^. Other targets and therapeutic approaches should therefore be considered. A temporally controlled, precisely targeted, and non-pharmacological approach to increase insulin secretion would be advantageous.

Neural interfacing, particularly at the peripheral nerve level, is increasingly studied and utilized as a mode of organ modulation for disease treatment^11,12^. Vagus nerve stimulation therapies have shown efficacy across numerous clinical areas, including inflammation in rheumatoid arthritis^13^, epileptic seizures^14^, depression^15^, obesity^16^, and migraine^17^. In all cases, however, the underlying neural mechanisms are poorly understood for at least two reasons. First, the electrical stimulation lacks axon-level specificity. Thus, activation of off-target pathways is unavoidable. For example, in animal models, while insulin is increased with electrical vagal stimulation, glucose remains unchanged or even increases^1–3,6^. Given the established understanding that insulin acts to decrease circulating glucose, this demonstrates that counteracting (off-target) pathways likely interfere with a signaling pathway of interest. Second, the neural circuitry is complex with many interacting components in both the periphery and brainstem. These extensive interactions can make it difficult to analyze and explain experimental results.

Highly specific activation of neural pathways is possible by means of optogenetics, whereby a light-activatable ion channel or pump can be genetically targeted to specific cell types. While this optical approach is genetically defined, it can achieve further specificity based on spatial shaping of the excitation light^18^. Here, we demonstrate light-activated stimulation of the endocrine pancreas by targeting parasympathetic axons using a transgenic mouse line expressing ChR2 under control of the choline acetyltransferase promoter which will be referred to as ‘ChAT-ChR2’. Cholinergic fibers were stimulated in two locations: first, stimulation of cholinergic axons and terminals in the pancreas was expected to activate pancreatic cholinergic efferents to cause insulin release. This was predicted to have few, if any, off-target consequences and to produce both insulin release and a reduction in blood glucose. However, we anticipated that laser stimulation via an ultrasound-guided cannula was unlikely to activate the entire pancreas. Second, stimulation of the cervical vagus nerve was hypothesized to cause two possible outcomes: (1) by activating all cholinergic axons of the pancreas, a more robust release of insulin and a decrease in blood glucose, relative to direct pancreas stimulation, would be observed or (2) insulin and glucose changes would be similar to electrical stimulation because we would be activating cholinergic efferents to all the thoracic and visceral organs. Our results showed significant reduction in glycemic levels when stimulating within the pancreas, in both basal and elevated glucose conditions. However, glycemia was not significantly reduced by cervical vagus nerve stimulation despite a significant increase in insulin secretion. Additionally, pancreatic blood flow was increased by cholinergic stimulation in the pancreas, in further support of parasympathetic activation in these experiments.

## Research design and methods

### Animals

The use of animals was approved by the Institutional Animal Care and Use Committee (IACUC) at the University of Colorado, Anschutz Medical Campus, with accreditation by the Association for Assessment and Accreditation of Laboratory Animal Care (AAALAC). All experiments were performed in accordance with IACUC regulations under an approved protocol, and the study was carried out in compliance with Animal Research Reporting of In Vivo Experiments (ARRIVE) guidelines. ChAT-Cre mice (JAX, #006410) and Rosa-ChR2-YFP mice (JAX, #012569) were crossed and bred in-house to homozygosity—referred to as ‘ChAT-ChR2′. Age-matched wild-type C57Bl6 mice (JAX) were used as controls. Mice were fasted 6 h prior to each experiment. Each experimental group contained a roughly equal distribution of male and female mice, and sex-specific differences were not analyzed in this study.

### Direct pancreas stimulation

Mice were anesthetized using 1–3% isoflurane, and placed supine on a heating pad and monitored throughout the experiment with body temperature and vitals measurement. Cannula placement was guided by ultrasound imaging with a VEVO2100 small animal ultrasound machine (Visual Sonics). The pancreas was identified by location in relation to the spleen, kidney, and stomach, and by image texture. An optical cannula (CFMC12L20, Thorlabs) was guided into the abdomen within a 22G needle and directed to the pancreas. A 473 nm-wavelength solid-state laser (SLOC) was coupled to the optical cannula with an optical patch cable (200 µm core, 0.39 NA, M81L005, Thorlabs) and an interconnect (ADAF2, Thorlabs). The laser was controlled with a custom Arduino circuit board to output 5 ms pulses at 20 Hz. The continuous-wave (non-pulsed) power output at the tip of the cannula was 45 mW. Blood glucose was measured from tail vein samples using a glucose meter (Bayer Contour) every five minutes throughout the experiment. After a 20-min baseline period, laser stimulation of the pancreas was applied for 25 min. In the non-laser stimulated control groups, mice were anesthetized only.

Immediately following the laser stimulation, a single sample of blood (~ 50 μl) was collected into a heparin-coated collection tube. Insulin and glucagon concentration were assessed by ELISA (Mouse ultra-sensitive insulin ELISA, STELLUX Chemi Glucagon ELISA, both Alpco). Blood glucose measurements were collected for 15 min following stimulation in the basal-glucose state experiments and for 75 min post-stimulation in the glucose tolerance tests. For glucose tolerance testing, a 200 mg/ml glucose solution in PBS was injected intraperitoneally for a dose of 2 g/kg body-weight. Following stimulation, mice were monitored until full recovery.

Glucose levels were compared *within group* in the following separate experimental groups: (1) ChAT-ChR2 with laser stimulation, (2) ChAT-ChR2 without laser stimulation, (3) Wild-type mice with laser stimulation, and (4) Wild-type mice without laser stimulation. In-group glucose deviation from pre-stimulation baseline was calculated as mean glucose levels during stimulus and post-stimulus periods relative to mean baseline glucose. For the glucose tolerance test, mean integrated glucose response was compared between laser stimulated ChAT-ChR2 mice and non-laser stimulated ChAT-ChR2 mice, during both the stimulation period and post-stimulation period.

### Blood flow measurements

Blood flow in the pancreas was measured in response to direct pancreas stimulation (as described above) using contrast-enhanced ultrasound (CEUS) imaging, as previously described^19^. Briefly, acquisition settings of the MS250 linear array transducer were set at 10% transmit power, 18 MHz, standard beamwidth, contrast gain of 30 dB, 2D gain of 18 dB, with an acquisition rate of 26 frames per second. Microbubbles (MB; 3-4 µm size-isolated, Advanced Microbubbles Laboratories) were injected as a single 100 µl bolus (10 × 10^6^ MBs), into the lateral tail via a custom-made 27½ G winged infusion set. Following infusion, contrast intensity was measured and allowed to reach a steady state. A high mechanical-index flash-destruction was then initiated within a region of interest of the pancreas (identified using B-mode imaging, see above). Recovery of contrast signal within this region was measured as a time-course averaged over the pancreas, normalized to the contrast signal over the last five seconds prior to flash-destruction. The rise rate of the reperfusion was determined through an exponential fit. Reperfusion rate values were compared *within group* in the following experimental groups: (1) ChAT-ChR2 with laser stimulation, (2) Wild-type mice with laser stimulation.

### Cervical vagus stimulation

Vitals were monitored with a MouseOx Plus suite (Starr Life Sciences). A 1–1.5 cm incision was made at the cervical region, 2–3 mm left of midline. Blunt dissection techniques were used to expose the left cervical vagus nerve and separate it from the carotid artery. The optical cannula was positioned with a micromanipulator to abut the nerve for laser stimulation. Blood samples were collected for glucose and insulin measurement as described above, once the surgical opening was complete. The non-laser stimulated control groups were given a sham surgical procedure, as described above but without turning the laser on. The laser pulsing was the same as in the direct pancreas stimulation (5 ms pulse duration, at 20 Hz). The continuous-wave (non-pulsed) power at the output of the cannula was 36mW. A downward slope in glucose during the procedure was slope-detrended using the linear regression of the non-laser-stimulated ChAT-ChR2 group. This slope was subtracted from all three experimental groups in Fig. 3 (non-detrended data is shown in Supplementary Fig. 5). In the current study we did not perform glucose tolerance tests during the more technically challenging cervical stimulation procedure.

### Statistical comparison

Differences between group means were tested with two-tailed t-tests. A paired t-test was employed for non-independent data and a two-sample unpaired t-test was used for independent group data. All error bars depict the standard error of the mean.

### Data and resource availability

The datasets generated and analyzed during the current study are available from the corresponding author upon reasonable request. No applicable (non-commercially available) resources were generated or analyzed during the current study.

## Results

### Direct pancreas cholinergic stimulation impacts basal glucose homeostasis

**Figure 1:**
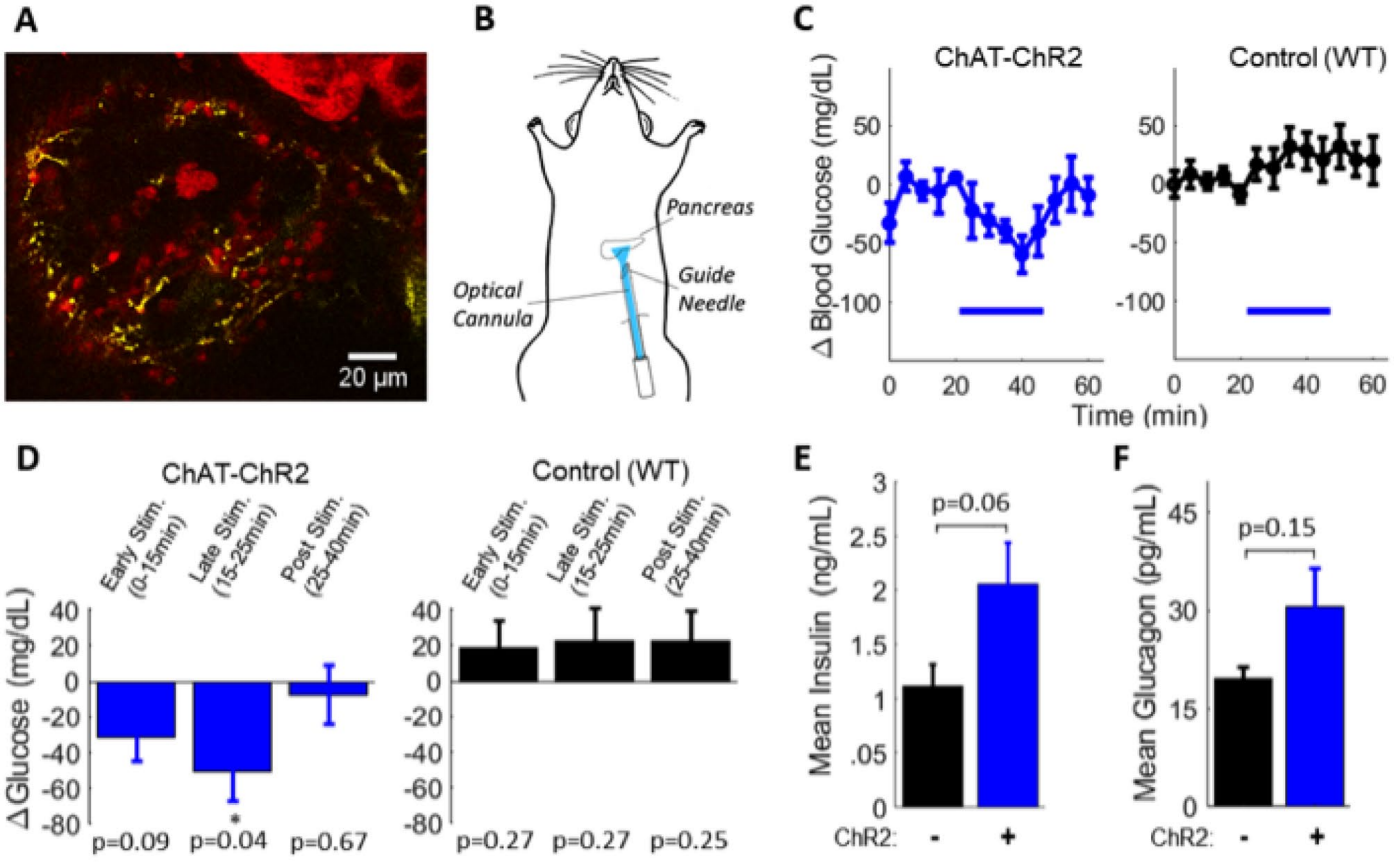
Direct optogenetic control of pancreas innervation at basal glucose. (**A**) Confocal section of a pancreatic islet within a tissue slice from a ChAT-ChR2 mouse, showing YFP fluorescence indicating regions of ChR2 expression in axon/terminals (yellow), and surrounding tissue labeled with Rhod-2 AM (red). (**B**) Diagram of optical stimulation at the pancreas in the anesthetized mouse. (**C**) Delta blood glucose in ChAT-ChR2 mice (n = 5) and wild-type mice (n = 5) experimental groups in response to optical stimulation (blue bar, 473 nm, 5 ms pulses, 20 Hz), and following stimulation. Baseline is the average of the prestimulus measurements. (**D**) Quantification of blood glucose changes during the early stimulation period (0–15 min from onset of stimulation), late stimulation period (15–25 min), and post stimulation period (25–40 min) in ChAT-ChR2 mice and wild-type mice. P-values refer to comparison with respect to pre-stimulus level. *Indicates statistically significant difference by student’s t-test. (**E**) Post-stimulation mean insulin levels of the two experimental groups sampled immediately following laser stimulation. (**F**) Post-stimulation mean glucagon levels of the two experimental groups sampled immediately following laser stimulation. Data in (**C**–**F**) presented as the mean over n = 5 ChaT-ChR2 recordings and WT recorisngs. Error bars represent standard error in the mean.

We first tested whether insulin and glucose could be modulated by direct pancreatic stimulation of cholinergic axons. Confocal imaging of YFP in pancreas slices from ChAT-ChR2 mice confirmed the presence of ChR2-expressing cholinergic axons that terminated within islets (Fig. 1A). In fasted mice (6 h), an optical cannula was ultrasound-guided to the pancreas tail (Fig. 1B, see also Fig. 4A). Glycemic levels were analyzed *within* group: glucose levels in ChAT-ChR2 mice rapidly decreased upon laser stimulation and recovered to baseline following stimulation. Wild-type (WT) control mice did not exhibit any stimulation-dependent decreases (Fig. 1C and Supplementary Fig. 1A,B). Blood glucose in ChAT-ChR2 mice was significantly reduced by − 50 mg/dl (− 34%) compared to baseline levels in the late stimulation period (15–25 min, p = 0.04), while glucose levels were not significantly changed in the wild-type control group (Fig. 1D). Mean insulin in the ChAT-ChR2 group was higher, but with borderline significance (+ 84%, p = 0.06) compared to the WT controls following laser stimulation (Fig. 1E). We note larger samples in future studies will help clarify this measurement. Laser stimulation had no impact on insulin levels in WT controls (Supplementary Fig. 1C). Mean glucagon in the ChAT-ChR2 group was not significantly different (+ 56%, p = 0.15) than in WT controls (Fig. 1F).

### Direct pancreas cholinergic stimulation affects glucose excursions

**Figure 2:**
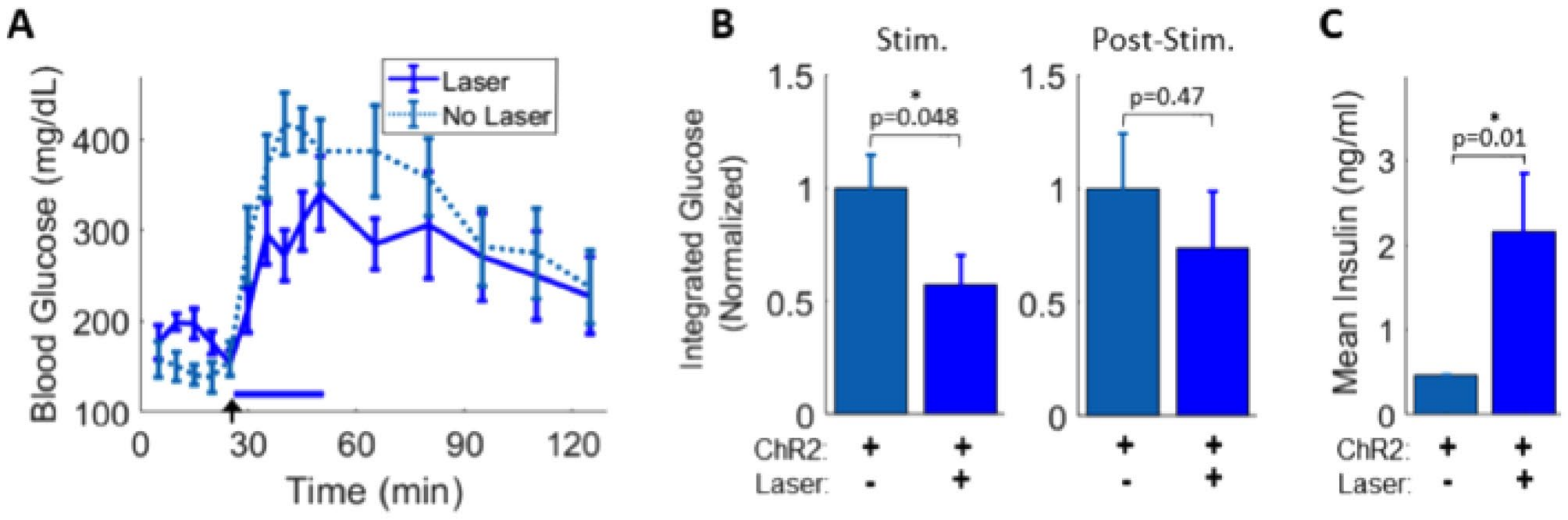
Direct optogenetic control of pancreas innervation following glucose elevation. (**A**) Blood glucose response in ChAT-ChR2 mice with and without laser stimulation (blue bar, 473 nm, 5 ms pulses, 20 Hz) following intraperitoneal glucose bolus injection (black arrow). (**B**) Integrated area under the glucose response curve during the stimulation period (left) and during the post-stimulation period (right). (**C**) Blood insulin concentration sampled immediately following stimulation is significantly increased (~ twofold; p = 0.01) in the laser-stimulated ChAT-ChR2 group relative to non-laser-stimulated ChAT-ChR2. Data in A-C presented as the mean over n = 8 recordings (with light) and n = 7 recordings (without light). Error bars represent standard error in the mean.

We next applied optogenetic stimulation to the pancreas during acute glucose elevations. Following glucose delivery, glycemic levels were blunted during laser stimulation (− 42%, p = 0.048), but not after laser stimulation (− 26%, p = 0.47) relative to non-laser-stimulated ChAT-ChR2 mice (Fig. 2A,B). WT control mice did not exhibit stimulation-dependent decreases in glucose levels (Supplementary Fig. 2A,B). Insulin levels were also significantly increased in laser-stimulated ChAT-ChR2 mice compared to non-laser-stimulated ChAT-ChR2 mice (+ 363%, p = 0.01) (Fig. 2C). In the absence of laser stimulation ChAT-ChR2 mice showed elevated glucose excursions relative to WT mice (Supplementary Fig. 3), thus preventing a direct comparison between laser-stimulation in ChAT-ChR2 and WT control mice.

### Cervical vagus cholinergic stimulation impacts insulin secretion but not blood glucose

**Figure 3:**
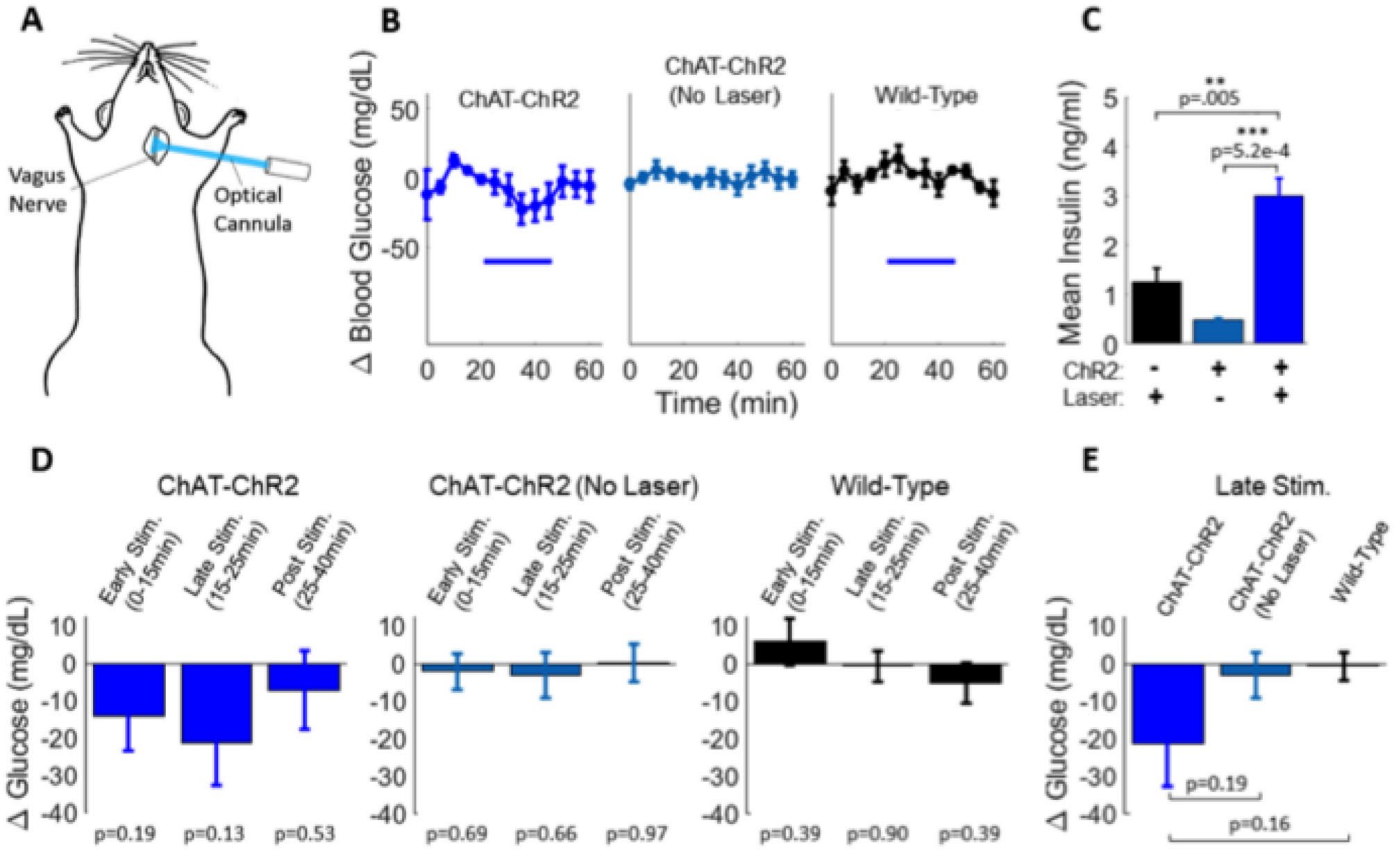
Cervical vagus nerve stimulation in the basal glucose state. (**A**) Schematic diagram of the location of laser stimulation. (**B**) Blood glucose levels in laser-stimulated ChAT-ChR2 mice (left, n = 6), non-laser-stimulated ChAT-ChR2 mice (middle, n = 6) and laser-stimulated wild-type mice (right, n = 5) (laser stimulation indicated by blue bar, 5 ms pulses, 20 Hz). (**C**) Quantification of post stimulation insulin levels showed a large, significant increase in the laser-stimulated ChAT-ChR2 group relative to controls. (**D**) Quantification of glucose changes in the early stimulation period (0-15 min following stimulation onset), late stimulation period (15–25 min) and post stimulation period (25–40 min). P-values refer to comparison with respect to pre-stimulus level. (**E**) Comparison of glucose changes during the late stimulation period across the three groups. Data in (**B**–**E**) presented as the mean over n = 6 ChaT-ChR2 recordings (with light and without light) and n = 5 WT recordings. Error bars represent standard error in the mean.

To test whether similar modulation of insulin secretion and glucose homeostasis could be achieved by proximal nerve stimulation, we stimulated cholinergic axons of the left cervical vagus nerve. Mice were surgically opened in the neck to allow vagus nerve exposure and optical stimulation (Fig. 3A). Laser stimulation of the cervical vagus nerve resulted in minor decreases in glucose that returned to baseline following stimulation (Fig. 3B). However significantly higher insulin levels were observed compared to wild-type controls (+ 140%, p = 0.005) and non-laser stimulated ChAT-ChR2 mice (+ 512%, p = 0.0005) (Fig. 3C). Discrepancy between wild-type controls and non-laser stimulated ChAT-ChR2 may be related to perturbed glucose tolerance that occurs in animals expressing ChR2 (Supplementary Fig. 3) and other optogenes^20^. Mean blood glucose in the ChAT-ChR2 group was reduced by − 23% relative to baseline in the late-stimulation period, although was not statistically significant (10–25 min, p = 0.13) (Fig. 3D). The mean reduction over this late-stimulation period was 20% lower compared to the non-laser-stimulated group (p = 0.19) and 23% lower relative to the wild-type group (p = 0.16) (Fig. 3E), but these differences were also not statistically significant.

### Cholinergic stimulation increases pancreatic blood flow

**Figure 4:**
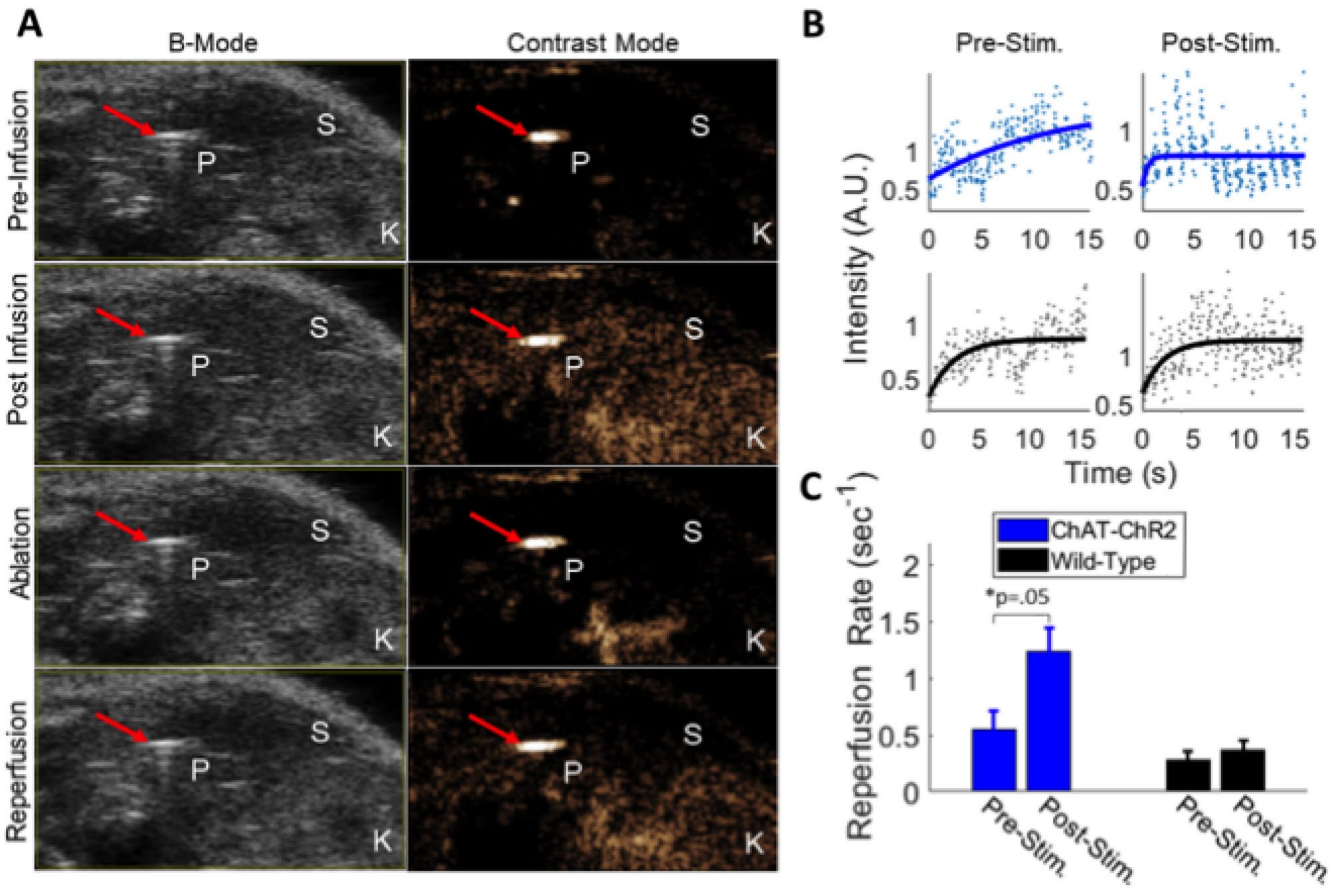
Measurement of pancreatic blood perfusion using contrast enhanced ultrasound imaging of microbubbles. (**A**) B-mode and sub-harmonic contrast mode images in the abdominal cavity. Microbubble-generated sub-harmonic contrast is observed in the tissues during microbubble infusion and during reperfusion following flash-destruction. Red arrow = optical cannula/needle tip, P = pancreas (tail), S = spleen, K = kidney. (**B**) Example single recording reperfusion data and curve fit before and after laser stimulation for a ChAT-ChR2 mouse and wild-type mouse. Intensity is based on microbubble (MB)-generated sub-harmonic contrast as MBs perfuse back into the pancreas following flash destruction. Curves are fitted to a single component inverse exponential to extract the rate of signal elevation (k_r_ = 1/time constant of recovery). Upper panels (blue points and curves) are from a ChAT-ChR2 mouse and lower panels (black points and curves) are from a wild-type mouse. (**C**) Mean reperfusion rate in ChAT-ChR2 mice and wild-type mice, prior to laser stimulation and after 25 min of laser stimulation. Data in C presented as the mean over n = 8 ChaT-ChR2 recordings and n = 5 WT recordings. Error bars represent standard error in the mean.

Parasympathetic activity has been suggested to modulate pancreas and islet blood flow^21,22^. Therefore, we tested whether optogenetic cholinergic stimulation would modulate pancreas blood flow. We quantified pancreas vascular perfusion dynamics using contrast-enhanced ultrasound following direct pancreas laser stimulation in 6 h fasted mice (Fig. 4A). Following flash-destruction of microbubbles within the pancreas, their recovery kinetics were measured before and after laser stimulation (Fig. 4B). The mean reperfusion rate increased significantly in ChAT-ChR2 mice (+ 123%, p = 0.05) indicating more rapid blood flow, while the wild-type group had no change in reperfusion rate following stimulation (p = 0.56) (Fig. 4C).

## Discussion

Electrical stimulation of the vagus nerve can stimulate endocrine pancreas hormone secretion, particularly insulin secretion. However reductions in blood glucose are not generally achieved^1–3,5,6^. The relatively indiscriminate electrical stimulation likely activates off-target pathways such as sensory and motor axons that innervate other organs. In the present study, we employed an optogenetic technique which enabled the optical stimulation of only cholinergic parasympathetic (and thus primarily efferent) axons. With this optical approach, insulin secretion was elevated in response to stimulation at either the pancreas or cervical vagus nerve. Importantly, and in contrast to electrical stimulation approaches, blood glucose was rapidly reduced during the pancreatic optical stimulation, under both basal glucose and glucose-elevated conditions. Blood flow was also increased in the pancreatic vasculature under pancreatic optical stimulation.

As cholinergic axons are broadly (but not exclusively) efferent, a likely explanation for the significant glucose reduction under optical stimulation of the pancreas is the omission of a large portion of afferent fiber activation and efferent to other organs that would occur under electrical stimulation. Vagus nerve afferent stimulation increases blood glucose in rats^6^. Furthermore, at basal glucose levels hepatic vagal afferents have been suggested to inhibit the brainstem centers which drive the efferent insulin secretory system^23^. Thus, the more substantial afferent activation during electrical stimulation of the whole vagus nerve may dampen insulin action, resulting in higher blood glucose levels. Nevertheless, the (non-significant) elevation in glucagon release seen here may temper the reduction in glucose achieved from direct pancreas stimulation, where substantial insulin release is stimulated. We also cannot exclude the possibility of indirect elevation of GH or epinephrine contributing to a dampening effect on glucose reduction.

We observed a more pronounced reduction in blood glucose during direct pancreas stimulation compared to cervical vagus stimulation. This occurred despite insulin increasing robustly under cervical stimulation, and thus not as a result of less efficient nerve stimulation. We hypothesized that cervical vagal stimulation would have opposing actions on blood glucose levels. On one hand, stimulation of all vagal efferent axons to the pancreas would enhance the effects observed during direct stimulation, which does not stimulate the entire pancreas. On the other hand, activating off-target organs would reduce the effects compared to direct stimulation. Our observation that glucose changes were not significant for cervical stimulation implies that off-target effects dominated. This is consistent with prior studies: for example, the chemical activation of cholinergic neurons in the dorsal motor nucleus of the vagus (DMV), the same population of efferents activated by cervical vagus stimulation in the present study, increased insulin secretion but did not reduce blood glucose^7^. The left vagus nerve innervates almost all the thoracic and abdominal organs, and thus cervical stimulation is almost certainly activating other organs which are not activated under direct pancreas stimulation. One possibility is that this substantial off-target activation reduces the net insulin action and glucose uptake. The notion that off-target vagal pathways may confound glucose metabolism is supported by experiments which methodically lesioned vagal branches and show optimal blood glucose reduction not with the fully intact vagus nerve, but with a partially vagotomized system^24^. This underlines the need for further specificity in targeting to achieve therapeutically beneficial glycemic control. We also note that isoflurane, as with most anesthesia, can impact glucose handling^25^. Thus, future experiments will examine glucose changes in the awake, unanesthetized animal with the aid of chronically implantable optical fibers and nerve cuffs.. While cholinergic optical stimulation in the cervical vagus causes less afferent activation than electrical stimulation, it is likely that more afferents were stimulated in the cervical stimulation than the direct pancreas stimulation. For instance, some ChAT-expressing neurons are present in the nodose ganglion of ChAT-ChR2 mice (Supplementary Fig. 4) indicating the presence of afferent expression. Nevertheless, these results suggest that electrical stimulation may also be directly activating other efferent pathways that reduce insulin action and prevent efficient modulation of glycemia. This points to the advantage provided by optogenetic approaches and image-guided stimulation to study and control neural inputs at the organ level.

The observed increase in blood flow within the pancreas is expected from parasympathetic stimulation. Cholinergic stimulation has been suggested (but not quantitatively demonstrated) to increase blood flow in the pancreas, given that blocking cholinergic receptors with atropine caused ischemia in pancreatic capillary beds^21^. Under increased glucose levels, during which parasympathetic activity increases, more rapid islet blood flow occurs relative to decreased glucose levels^22,26^. Conversely, stimulation of adrenergic nerve fibers decreases pancreatic blood flow^5,26^. The increase in blood flow due to cholinergic fiber stimulation shown here, possibly via upstream vasodilation may contribute to increased insulin release, although precise links between islet blood flow and hormone secretion remain to be determined. For example, the blood flow changes upon cholinergic fiber stimulation may instead result from increased insulin release and co-release of vasodilators such as adenosine^26^. It is also plausible that cholinergic stimulation could increase vascular permeability and thus effective insulin output due to dilation of vascular pericytes^27^. We note that the current experimental design for both blood flow and hormone/glycemic measurement did not distinguish the relative proportion of pre-ganglionic cholinergic versus post-ganglionic cholinergic parasympathetic fibers that underwent stimulation. However, the increased blood flow argues against significant stimulation of pre-ganglionic cholinergic sympathetic fibers, given that sympathetic stimulation would decrease pancreas blood flow.

The results of this study, utilizing novel optogenetic and image-guiding approaches, demonstrate effective control of both hormone secretion and blood glucose levels, which is not observed with whole nerve electrical stimulation. This highlights the functional improvement that can be achieved by increasing neural specificity, and the potential of optogenetic interfaces towards this. This methodology will be valuable for studying signaling pathways within the endocrine pancreas in vivo, particularly those involving cellular excitability. While the reduction in blood glucose in response to cervical vagus stimulation was not statistically significant, despite robust insulin secretion, the efficacy of cervical stimulation will be improved by confining the specificity of opsin expression to pancreas-specific axons. Future studies should therefore investigate the use of a pancreas-injected retrograde AAV^28^ to transduce opsin expression in pancreatic axons, as well as tighter genetic control for appropriate specificity.

Translation of these techniques to human testing may rest on further elucidation of the degree of insulin secretion that can be impacted by neural inputs. It has been reported in humans that cholinergic activation of β-cell insulin secretion occurs as a paracrine signal from α-cells which release acetylcholine^29,30^. However, cholinergic neural inputs do exist in human islets, although to a lesser extent than in rodent islets^31–33^. In humans, a complex picture is emerging of intra-islet cellular communication and signaling of hormone secretion^29,30,34^. There is evidence that γ-cells may receive neural input, and interestingly these cells contain the highest expression levels of muscarinic cholinergic receptors^35^. As many neurotransmitters can function as paracrine signals within the human islet^34^, a paracrine signal originating from axon terminals influencing insulin secretion should not be ruled out. Whether or not neural processes directly innervate insulin-secreting β-cells, the function of neural processes within the highly interdependent network of islet cells needs to be better understood before clinical testing is warranted.

Delivery of opsins in humans for targeted optogenetic stimulation may be feasible with AAV constructs. AAVs are widely used to deliver optical actuators and sensors in experimental models, with targeting specificity defined by tissue/location of delivery, virus serotype, and genetic promoter. There are numerous AAV-based gene therapies in human clinical trials, and to date, three have been approved for treatment in the U.S. and E.U.^36–39^. Additional steps toward a potential clinically translatable therapy include the integration of an implanted nerve cuff to allow stimulation in awake and behaving subjects, combined with continuous glucose monitoring and closed-loop control.

## Supplementary Information

Supplementary Information.
