# Supplementary material for: Optogenetic stimulation of cholinergic fibers for the modulation of insulin and glycemia
Source: Sci Rep. 2021 Feb 11;11:3670. doi: 10.1038/s41598-021-83361-3 (PMC7878862; doi:10.1038/s41598-021-83361-3)
Supplement: Supplementary file 1 — Supplementary Information. [file 41598_2021_83361_MOESM1_ESM.docx]

**Optogenetic Stimulation of Cholinergic Fibers for the Modulation of Insulin and Glycemia**

Arjun K. Fontaine^1,3*^, David G. Ramirez^1,4^, Samuel F. Littich^1,3^, Robert A. Piscopio^4^, Vira Kravets^4^, Wolfgang E. Schleicher^4^, Naoko Mizoguchi^5^, John H. Caldwell^2†^, Richard F. ff. Weir^1,3†^, Richard K.P. Benninger^1,4†^

Departments of ^1^Bioengineering, ^2^Cell and Developmental Biology, and the ^3^Biomechatronics Development Laboratory - University of Colorado, Anschutz Medical Campus.

^4^Barbara Davis Center for Childhood Diabetes – Anschutz Medical Campus

Division of ^5^Pharmacology, Department of Diagnostic and Therapeutic Sciences, Meikai University School of Dentistry, Saitama, Japan

*Correspondence to Arjun K. Fontaine or Richard K.P. Benninger

Department of Bioengineering, University of Colorado – Anschutz Medical Campus

[*arjun.fontaine@cuanschutz.edu*](mailto:arjun.fontaine@cuanschutz.edu), richard.benninger@cuanschutz.edu

*Ph: (303) 875 7064*

† Co-senior Authors: Richard K.P. Benninger, Richard F. *ff.* Weir, John H. Caldwell

**
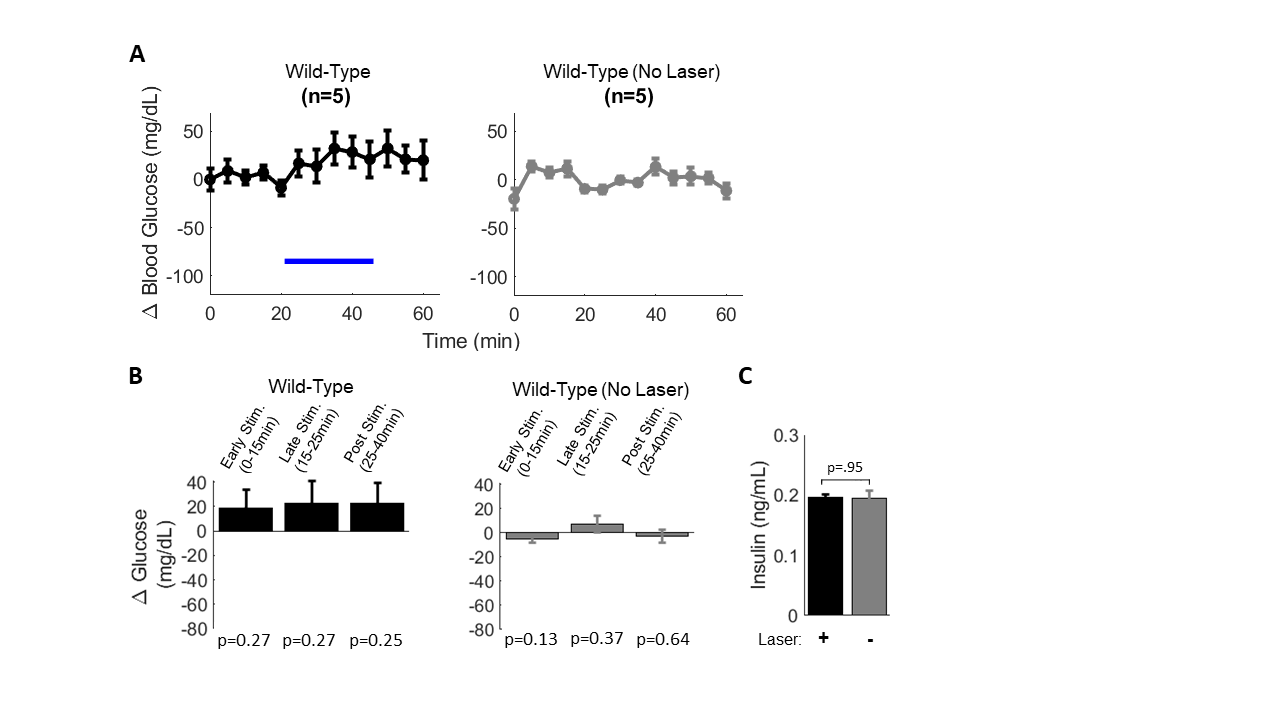
**

**Supplementary Figure 1:** Glucose and insulin measurements in wild-type control mice with and without direct pancreas optical stimulation. (A) Mean change in glucose (delta glucose) values throughout the experiment. (B) Delta glucose quantification shows no significant glucose changes in either group. (C) Mean insulin levels are not different between groups. Error bars represent standard error in the mean.


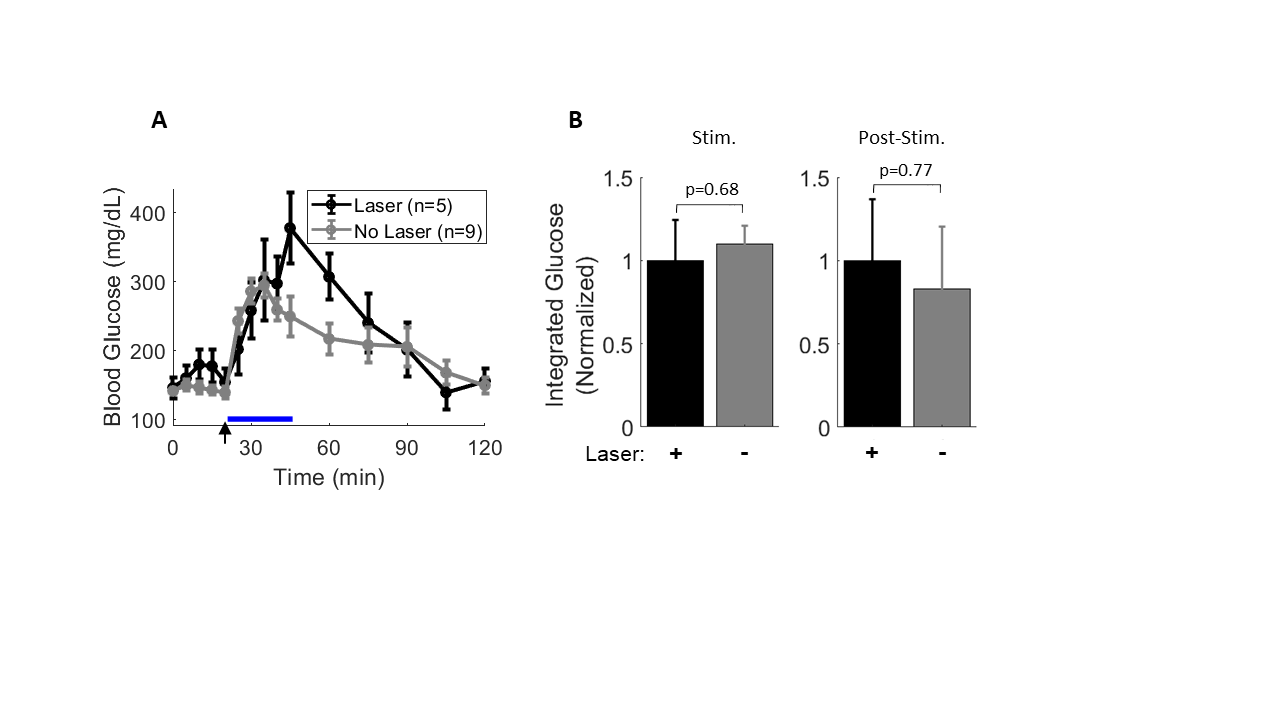


**Supplementary Figure 2:** Glucose tolerance tests in wild-type mice with and without laser direct pancreas optical stimulation. (A) Mean glucose levels over the experiment in which i.p. bolus injection of glucose is administered (black arrow). (B) Integrated glucose during (left) and after (right) stimulation shows no significant difference between groups. Error bars represent standard error in the mean.


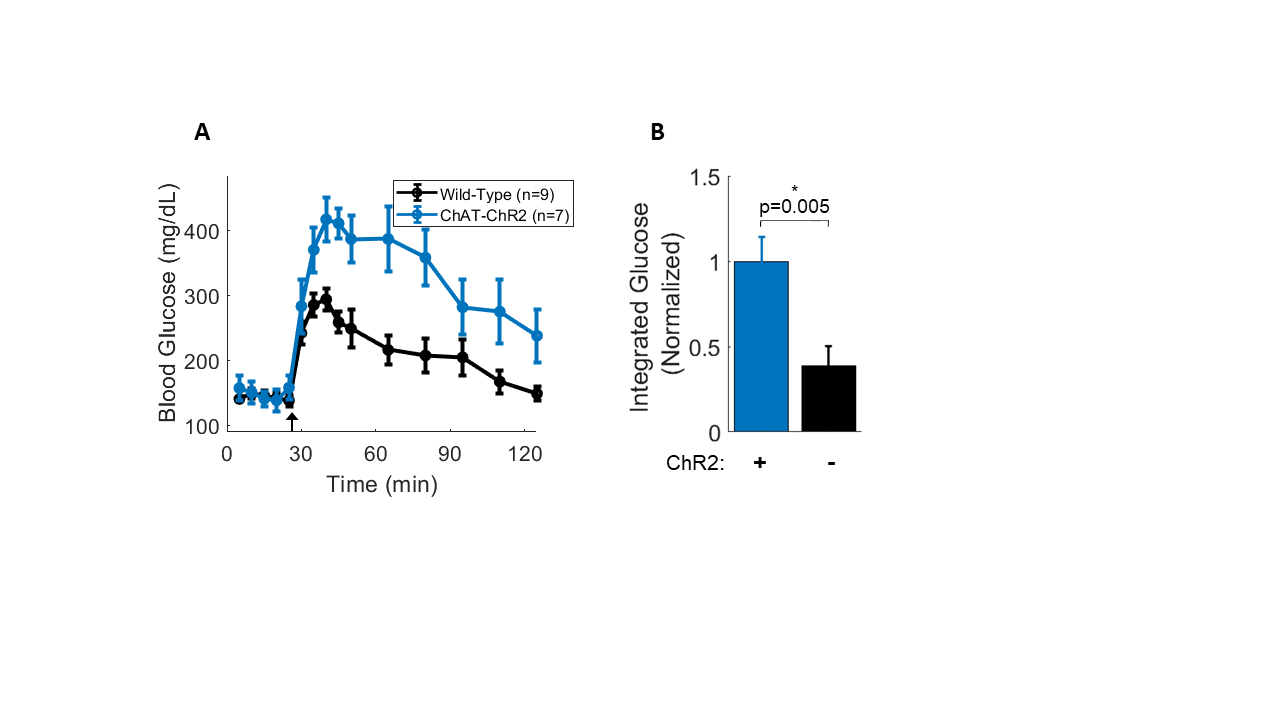


Supplementary Figure 3: Glucose tolerance tests without optical stimulation in ChAT-ChR2 and wild-type mice. (A) Mean glucose levels over the experiment in which i.p. bolus injection of glucose is administered (black arrow). (B) Integrated glucose from the time of glucose injection to the end of the experiment shows a significant difference between the groups. Error bars represent standard error in the mean.


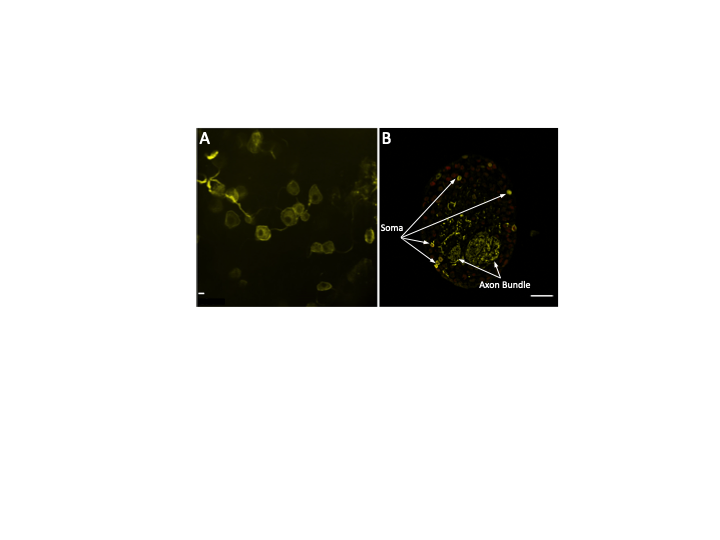


Supplementary Figure 4: (A) Confocal image in nodose ganglion of a ChAT-ChR2-YFP mouse showing ChR2-YFP expression in neurons within the ganglion (scale bar, 10 μm). (B) Axial section of the nodose ganglion showing sparse neuronal labeling, and fasciculated cholinergic vagal axon bundles (scale bar, 100 μm).


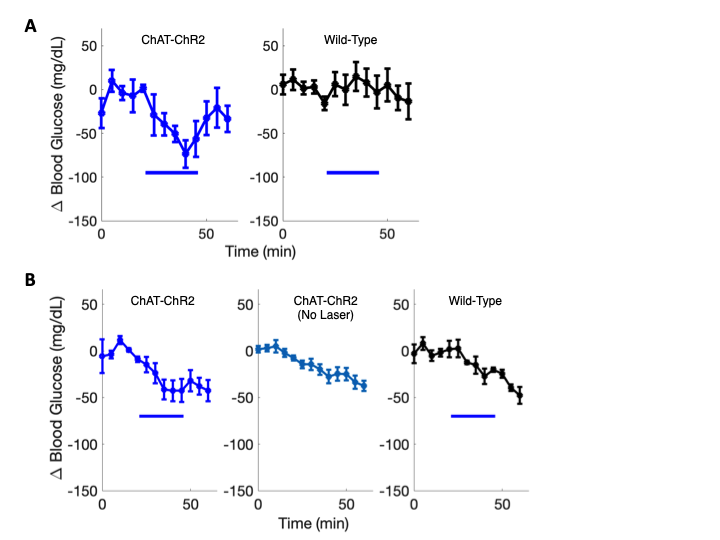


**Supplementary Figure 5:** (A) Non-detrended glucose for direct pancreas stimulation experiments (Figure 1C). Mean baseline glucose was 217±43 (mg/dL) for the Chat-ChR2 group and 172±11 (mg/dL) for the Wild-Type group. (B) Non-detrended glucose for cervical vagus nerve stimulation experiments (Figure 3B). Mean baseline glucose was 154±20 for the ChAT-ChR2 group, 146±19 for the ChAT-ChR2 (No Laser) group, and 188±39 for the Wild-Type group.
